# L’occlusion intestinale aigue gravidique - à propos de cinq cas

**Published:** 2012-03-07

**Authors:** Mohammed Najih, Mohamed Abdellaoui, My Rachid Hafidi, Hicham Laraqui, Sifeddine AlKandry

**Affiliations:** 1 Service de chirurgie viscérale, Hôpital Militaire d’Instruction Mohamed V, Rabat, Maroc; 2 Service de gynécologie-obstétrique, Hôpital Militaire d’Instruction Mohamed V, Rabat, Maroc

**Keywords:** Occlusion intestinale, grossesse, chirurgie, traitement conservateur

## Abstract

L’occlusion intestinale survient rarement au cours de la grossesse mais elle s’accompagne d’une morbidité et mortalité maternelle et fœtale élevées souvent dues au retard diagnostic et thérapeutique. Les auteurs rapportent cinq cas d’obstruction intestinale gravidique, et revoient la littérature afin de discuter les mécanismes physiopathologiques, les difficultés diagnostiques et thérapeutiques et de proposer une conduite à tenir adéquate.

## Introduction

Une occlusion intestinale complique rarement une grossesse, l’errance diagnostique, la réticence des cliniciens à prescrire des examens radiologiques chez une femme enceinte et le retard thérapeutique sont les principaux facteurs de morbi-mortalité maternelle et fœtale qui restent élevées [1]. C’est à travers cinq observations d’occlusion intestinale gravidique et une revue de la littérature que nous discutons la physiopathologie et les problèmes diagnostiques et thérapeutiques de cette entité.

## Méthodes

Il s’agit d’une étude rétrospective des cas d’occlusion intestinale survenant au cours de la grossesse colligés dans le service de chirurgie viscérale à l’Hôpital Militaire d’instruction Mohamed V, durant une période allant de 2000 à 2011. Durant cette période, cinq patientes enceintes étaient admises pour occlusion intestinale (les différentes observations sont illustrées dans le Tableau 1).

**Tableau 1:** Tableau récapitulatif des différentes observations de l’occlusion aigue et grossesse

	Cas 1	cas 2	cas3	Cas4	Cas5
Age	42 ans	34 ans	27 ans	38 ans	23 ans
ATCDS obstétricaux	G2P2	G2P0	G1P0	G3P2	G1PO
ATCDS chirurgicaux	−	appendicectomie	Pancréas annulaire	Péritonite appendiculaire	−
AG(SA)	16	17	35	35	37
Signes cliniques	Vomissementsdouleurs ab dAMG	Vomissementsdouleurs ab d	Vomissementsdouleurs ab dAMG	Vomissementsdouleurs ab dAMG	Vomissementsdouleurs ab dAMG
Biologie	GB : 11300/mm^3^	GB : 15800/mm^3^	GB : 15100	GB : 16300	GB : 37000
ASP	N H A	N H A	−	−	arceau gazeux refoulé par l’opacité utérine (figure 2)
Echographie	dilatations des anses; grossesse évolutive	dilatations des anses; grossesse évolutive	dilatations des anses; grossesse évolutive	progression de la dilatation des anses (figure 1); grossesse évolutive	grossesse non évolutive
Retard de Diagnostic	7 jours	6 heures	2 jours	2 jours	3 jours
Traitement Conservateur	Non	24 heures	5jours	24 heures	non
Cause de L’occlusion	tumeur du colon gauche	Bride	bride avec nécrose grélique (30 cm)	Adhérences	volvulus du sigmoïde avec nécrose (figure3)
Méthodes Chirurgicale	stomie de proche amant	section de la bride+adhésiolyse	résection de 30 cm du grêle + adhésiolyse	adhésiolyse	résection du sigmoïde+double stomie
Tocolyse	+	+	−	−	−
Devenir fœtal	césarienne à 40 SA n-né bien portant	à terme (38SA) n-née (apgar9-10)	césarienne, n-né apgar 6-9	césarienne pour souffrance fœtale; n-née 2622grames	mort-né

AG: âgé gestationnel ; SA : semaine d’aménorrhée; G : gestation ; P : parité ; NHA : niveau hydro-aérique; N-né : nouveau-né

## Résultats

**Figure 1:**
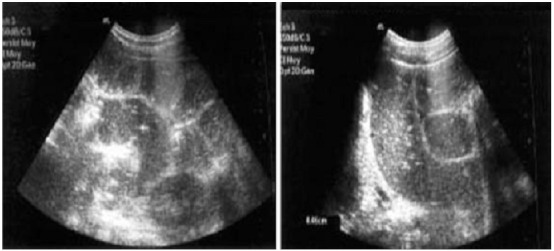
Echographies abdominales à 24 heures d’intervalle montrant une aggravation du degré de dilatation des anses grêles

**Figure 2:**
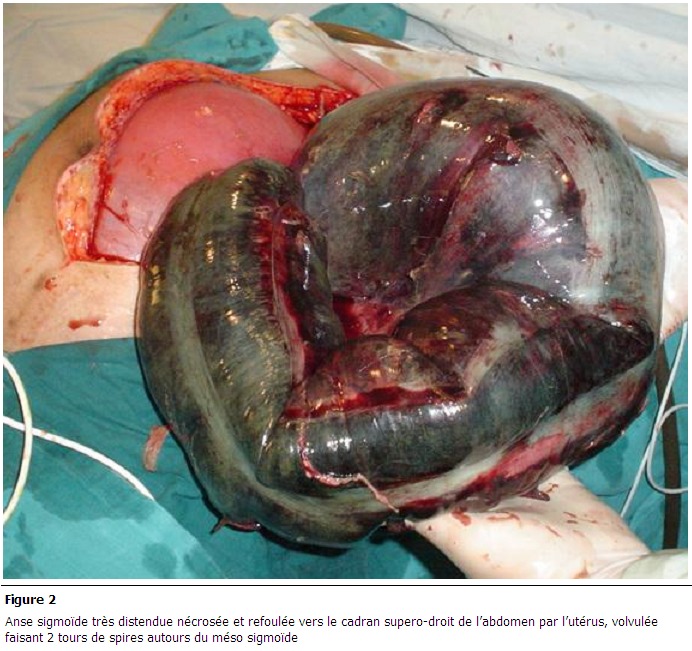
Anse sigmoïde très distendue nécrosée et refoulée vers le cadran supero-droit de l’abdomen par l’utérus, volvulée faisant 2 tours de spires autours du méso sigmoïde

La moyenne d’âge était de 30 ans, avec des extrêmes allant de 23 à 42 ans. L’âge gestationnel variait entre 16 SA et 37 SA (deux patientes au cours du 2éme trimestre et les autres au 3^ème^ trimestre). Parmi les cinq patientes trois avait des antécédents de chirurgie abdominale. Le retard de diagnostic allait jusqu’aux 7 jours. Le traitement conservateur était tenté chez 3 patientes au cours du troisième trimestre; il a permet d’atteindre le terme chez une seul patiente. Toutes les patientes ont subi une intervention chirurgicale. Une bride était la cause de l’occlusion chez trois cas, une tumeur sténosante du colon gauche était retrouvée dans un cas et un volvulus du sigmoïde dans un cas. Deux patientes ont reçu une tocolyse prophylactique, aucun effet indésirable de tocolyse n’a été noté. Trois patientes ont eu un accouchement par voie basse et les deux autres ont subi une césarienne ; l’une d’elles au cours de la même laparotomie pour occlusion. Un seul mort-né été noté ; les quatre autres bébés étaient en bonne santé (Tableau 2). Les Figure 1 et Figure 2 présentent des lésions retrouvées chez certaines de nos patientes.

**Tableau 2:** Résumé des résultats

Age moyen	A.G.	ATCD	Signes cliniques	Examen radiologique	Indication thérapeutique	Devenir obstétricale
30 ans	2^ème^ tr.: 2 cas. 3^ème^ tr.: 3 cas	Chirurgical: 3 cas	Arrêt des matières des gaz: 4 cas Vomissement: 5 cas	Échographie: 5 cas. ASP: 5cas.TDM: 1 cas	Chirurgical: 5 cas	Bébé en bonne santé: 4 cas. Mort-né: 1 cas

Tr. : trimestre AG : âge gestationnel

## Discussion

L’occlusion intestinale survenant au cours de la grossesse est très rare [1,2] l’incidence varie entre 1/1.500 à 1/66.000 grossesses [3,4]. Environ 53-59% des obstructions intestinales sont dues à des adhérences ou brides secondaires à des interventions chirurgicales antérieures ou à des épisodes de maladies inflammatoires du pelvis [2–4].

au cours de la gestation, l’occlusion intestinale serait favorisée par la diminution du péristaltisme intestinale et l’augmentation de la constipation habituelle due à l’imprégnation progestative favorisant une hypotonie de la musculature lisse intestinale et par modification topographique des brides séquellaires d’une intervention antérieure suite au développement de l’utérus gravide [3,5] ; ainsi les auteurs décrivent trois périodes à risque élevé, entre 16 et 20 SA lorsque l’utérus devient abdomino-pelvien, au voisinage de la 36^ème^ SA lorsque la tête descend dans le pelvis et dans le post-partum immédiat par une diminution brutale du volume utérin [6,7].

Le diagnostic clinique est souvent difficile et tardif car les signe habituels de l’occlusion (douleurs, distension, vomissements, constipation) sont mis au compte des signes sympathiques de la grossesse ; en plus le déplacement des organes abdominaux au fur et à mesure que la grossesse progresse donne des localisations atypiques de la douleur. C’est dire l’importance de l’examen clinique et de l’observation attentive devant une telle symptomatologie [1]. Toutes nos patientes ont présenté des vomissements alors que l’arrêt des matières et des gaz était présent chez uniquement trois patientes. Les analyses biologiques sont souvent peu concluantes ; La grossesse s’accompagne d’une hyper leucocytose physiologique qui peut varier entre 9000 à 12000 en raison de l’augmentation de l’activité corticosurrénale et donc serait un indicateur peu fiable chez la femme enceinte [2,3,8]. Toutefois, une augmentation significative en quelques heures de la leucocytose doit attirer l’intention [9]. Chez nos patientes hormis une hyperleucocytose et un tableau clinique suspectant une occlusion aigue, une confirmation radiologique était nécessaire.

L’échographie abdominale constitue l’examen diagnostique de première intention en cas de suspicion d’obstruction intestinale au cours de la grossesse. Elle permet d’exclure le diagnostic avec une sensibilité de 89 % et une spécificité de 100 %. Son innocuité autorise des examens répétés qui permettent de suivre l’évolution de la dilatation des anses intestinales [6,8] ; comme c’est le cas pour la 4^ème^ observation (Tableau 1). Cette évolution permet d’évaluer la pertinence du traitement conservateur et constitue un élément déterminant pour la décision chirurgicale. L’échographie permet un bilan complet de la sphère abdominale et de la grossesse à la recherche d’autres pathologies. Le couple échographie abdominale informative et évaluation clinique était suffisant pour confirmer le diagnostic d’occlusion intestinale chez toutes nos patientes.

La radiographie d’abdomen sans préparation (ASP) (sensibilité : 75% et spécificité : 53%) est un second choix d’autant plus que les niveaux hydro-aériques (NHA) habituellement recherchés ne sont présents qu’au cours des premières heures lors de l’installation de l’occlusion intestinale, le versant aérique étant alors comblé par du liquide de stase intraluminale. Toutefois, cette imagerie reste utile dans le bilan général d’obstruction intestinale en cas d’échographie non conclusive et l’hésitation des cliniciens à prescrire cette imagerie chez la femme gravide par crainte d’induire des malformations fœtales est injustifiée en regard du risque de morbidité et de mortalité élevée si le diagnostic est retardé [9]. Les complications majeures qui résultent d’un diagnostic tardif sont l’ischémie intestinale et le choc septique, le taux de mortalité maternelle pouvant s’élever entre 6 à 20% [10]. L’ASP était réalisé chez toutes nos patientes, il a permet de montrer des NHA chez trois cas. La résonance magnétique nucléaire, si le centre en dispose peut être utilisée dans un cadre de diagnostic différentiel du fait de son innocuité pour le fœtus au-delà du premier trimestre et des informations qu’elle peut procurer [11]. Tandis que la tomodensitométrie n’est pas recommandée pendant la grossesse, sauf exceptionnellement lorsque l’avantage potentiel justifie le risque encouru pour le fœtus et au cas par cas, en étroite concertation avec l’obstétricien et le chirurgien digestif.

La majorité des observations de la littérature décrivent une pratique médicale visant à instaurer un traitement conservateur dans l’espoir d’éviter le traitement chirurgical. Cette attitude ne semble pas appropriée. La grossesse constituant en soi une cause complémentaire à l’obstruction, le traitement médical isolé se solde le plus souvent par un échec. Une intervention chirurgicale reste pratiquée dans 89 % des cas d’occlusion intestinale survenant durant la grossesse [3,4,12]. En retardant la prise en charge chirurgicale, cette attitude contribue cependant à l’élévation des mortalités maternelle et fœtale [13]. Trois de nos patientes (cas 2, 3 et 4) ont subi un traitement conservateur mais l’indication opératoire n’a pu être évité et une patiente (cas 3) avait une nécrose grélique ayant nécessité une résection anastomose probablement due en partie au retard de la chirurgie.

La base du traitement de l’occlusion est la chirurgie au temps opportun quelque soit le terme de la grossesse pour améliorer le pronostic et éviter les complications redoutables [14]. Le principe du traitement varie en fonction de l’âge gestationnel: Jusque 26 semaines: laparotomie avec levée de l’occlusion, poursuite de la grossesse jusqu’à son terme si possible ; entre 26 et 34 semaines : si possible la maturation pulmonaire fœtale suivie de césarienne avec incision cutanée médiane complétée d’un traitement chirurgical de l’occlusion ; entre 34 semaines et le terme : césarienne avec incision cutanée médiane complétée d’un traitement chirurgical de l’occlusion ; dans tous les cas, laparotomie ou césarienne impérativement endéans les 72 heures.

La préparation préopératoire des patientes doit nécessiter une décision collégiale incluant un obstétricien, un réanimateur-anesthésiste et un chirurgien afin de discuter au cas par cas la tocolyse prophylactique, la corticothérapie pour la maturation fœtale au troisième trimestre et l’indication chirurgicale.

Le pronostic materno-fœtal est fonction de la rapidité diagnostic et de la précocité du traitement ; Harer a rapporté une mortalité maternel de l’ordre de 21 % avec une mortalité fœtale de 31% [15]. Le développement des moyens de surveillance obstétricale, une prise en charge chirurgicale précoce ont permis l’amélioration du pronostic maternel avec une mortalité devenue presque nul ; La mortalité fœtale a peu changé avec le temps, de l’ordre de 20 à 30%, probablement du en grande partie à la prématurité [16]. Dans notre série on a eu un mort né avec un pourcentage de 20%.

## Conclusion

Le diagnostic de l’occlusion intestinale au cours de la grossesse est souvent difficile et tardif car Les troubles digestifs sont souvent mis sur le compte de la grossesse ; toutefois leur persistance ou leur apparition après le 1er trimestre doit inquiéter le clinicien et l’inciter à demander les examens complémentaires adéquats. Une prise charge multidisciplinaire et une chirurgie à temps est nécessaire pour minimiser la morbidité et la mortalité maternelles et fœtales.

## References

[1] Kalu E, Sherriff E, Alsibai MA, Haidar M (2006). Gestational intestinal obstruction: a case report and review of literature. Arch Gynecol Obstet..

[2] Chang YT, Huang YS, Chan HM, Chan HM, Huang CJ, Hsieh JS, Huang TJ (2006). Intestinal obstruction during pregnancy. Kaohsiung J Med Sci.

[3] Perdue PW, Johnson HW, Staffort PW (1992). Intestinal obstruction complicating pregnancy. Am J Surg..

[4] Goldthrop WO (1966). Intestinal obstruction during pregnancy and puerperium. Br J Clin Pract.

[5] Beck WW (1974). Intestinal obstruction in pregnancy. Obstet Gynecol..

[6] Bourque MR, Gibbons JM (1979). Intussusception causing intestinal obstruction in pregnancy. Conn Med..

[7] Scheible W, Goldbergre LE (1979). Diagnosis of small bowell obstruction: The contribution of diagnostic ultrasound. AJR..

[8] Musoke F, Kawooya MG, Kiguli-Malwadde E (2003). Comparison between sonographic and plain radiography in the diagnosis of small bowel obstruction at Mulago Hospital, Uganda. East Afr Med J.

[9] Connolly MM, Unti JA, Nora PF (1995). Bowel obstruction in pregnancy. Surg Clin North Am..

[10] Watanabe S, Otsubo Y, Shinagawa T, Araki T (2000). Small bowel obstruction in early pregnancy treated by jejunotomy and total parenteral nutrition. Obstet Gynecol..

[11] Juglard R, Rimbot A, Marty A (2003). Bowel obstruction in pregnancy: value of Single Shot Fast Spin Echo MR sequence (SS-FSE). J Radiol..

[12] Meyerson S, Holtz T, Ehrinpreis M, Dhar R (1995). Small bowel obstruction in pregnancy. Am J Gastroenterol..

[13] Kang HJ, Kim SH, Ryu JH, Choi SJ, Roh CR (2011). A case of intussusception managed conservatively in pregnancy. J Womens Med..

[14] Kolusari A, Kurdoglu M, Adali E, Yildizhan R, Sahin HG, Kotan C (2009). Sigmoid volvulus in pregnancy and puerperium: a case series. Cases J.

[15] HARER WB, HARER WB (1958). Volvulus complicating pregnancy and puerperium; report of three cases and review of literature. Obstet Gynecol.

[16] Lazaro EJ, Das PB, Abraham PV (1969). Volvulus of the sigmoid colon complicating pregnancy. Obstet Gynecol..

